# Locked in a pro-inflammatory state

**DOI:** 10.7554/eLife.80699

**Published:** 2022-07-07

**Authors:** Chiu Wang Chau, Ryohichi Sugimura

**Affiliations:** 1 School of Biomedical Sciences, Li Ka Shing Faculty of Medicine, University of Hong Kong Hong Kong Hong Kong

**Keywords:** COVID-19, SARS-CoV-2, macrophage polarization, efferocytosis, hyperinflammation, tissue repair, Human

## Abstract

Macrophages absorbing cells infected with viable SARS-CoV-2 particles fail to transition into an anti-inflammatory state, potentially contributing to a damaging immune reaction linked to severe forms of COVID-19.

**Related research article** Salina ACG, Dos Santos D, Rodrigues TS, Fortes-Rocha M, Freitas-Filho EG, Alzamora-Terrel DL, Castro IMS, Fraga-Silva TF, de Lima MHF, Nascimento DC, Silva CM, Toller-Kawahisa JE, Becerra A, Oliveira S, Caetite DB, Almeida L, Ishimoto AY, Lima TM, Martins RB, Veras FP, do Amaral NB, Giannini MC, Bonjorno LP, Lopes MIF, Benatti MN, Batah SS, Santana RC, Vilar FC, Martins MA, Assad RL, deAlmeida SCL, de Oliveira FR, Arruda Neto E, Cunha TM, Alves-Filho JC, Bonato VLD, Cunha FQ, Fabro AT, Nakaya HI, Zamboni DS, Louzada-Junior P, de Oliveira RDR, Cunha LD. 2022. Efferocytosis of SARS-CoV-2-infected dying cells impairs macrophage anti-inflammatory functions and clearance of apoptotic cells. *eLife* **11**:e74443. doi: 10.7554/eLife.74443.

In the last two and a half years, scientists all over the world have worked relentlessly to develop treatments and vaccines against SARS-CoV-2, the virus causing COVID-19. While considerable progress has been made identifying key properties of the virus, several fundamental questions remain. For example, it is still unclear why some people develop long COVID-19, or why others are asymptomatic.

Previous research has shown that some patients with COVID-19 can experience a cytokine storm, which is characterized by a high concentration of pro-inflammatory proteins called cytokines (Wong, 2021; Ragab et al., 2020). Cytokine storms can be an indicator of a poor disease prognosis, and research indicates that they contribute to long-term, and sometimes life-threatening, conditions in patients with long COVID-19 (Rai et al., 2021). Now, in eLife, Larissa Cunha and colleagues at the Universidade de São Paulo – including Ana Salina, Douglas dos-Santos, Tamara Rodrigues, and Marlon Fortes-Rocha as joint first authors – report new insights into how COVID-19 may cause cytokine storms (Salina et al., 2022).

Immune cells called macrophages are the major cell type responsible for cytokine storms in COVID-19 (Merad and Martin, 2020). Typically, they migrate to infected or damaged sites in the body, and upon contact with bacteria, viruses, or chemicals emitted by dying cells, produce proinflammatory cytokines (Figure 1). These, in turn, strengthen the response of other immune cells. Once the pathogens have been eliminated, macrophages stop producing proinflammatory cytokines and instead start releasing anti-inflammatory signals, which promote healing.

**Figure 1.**
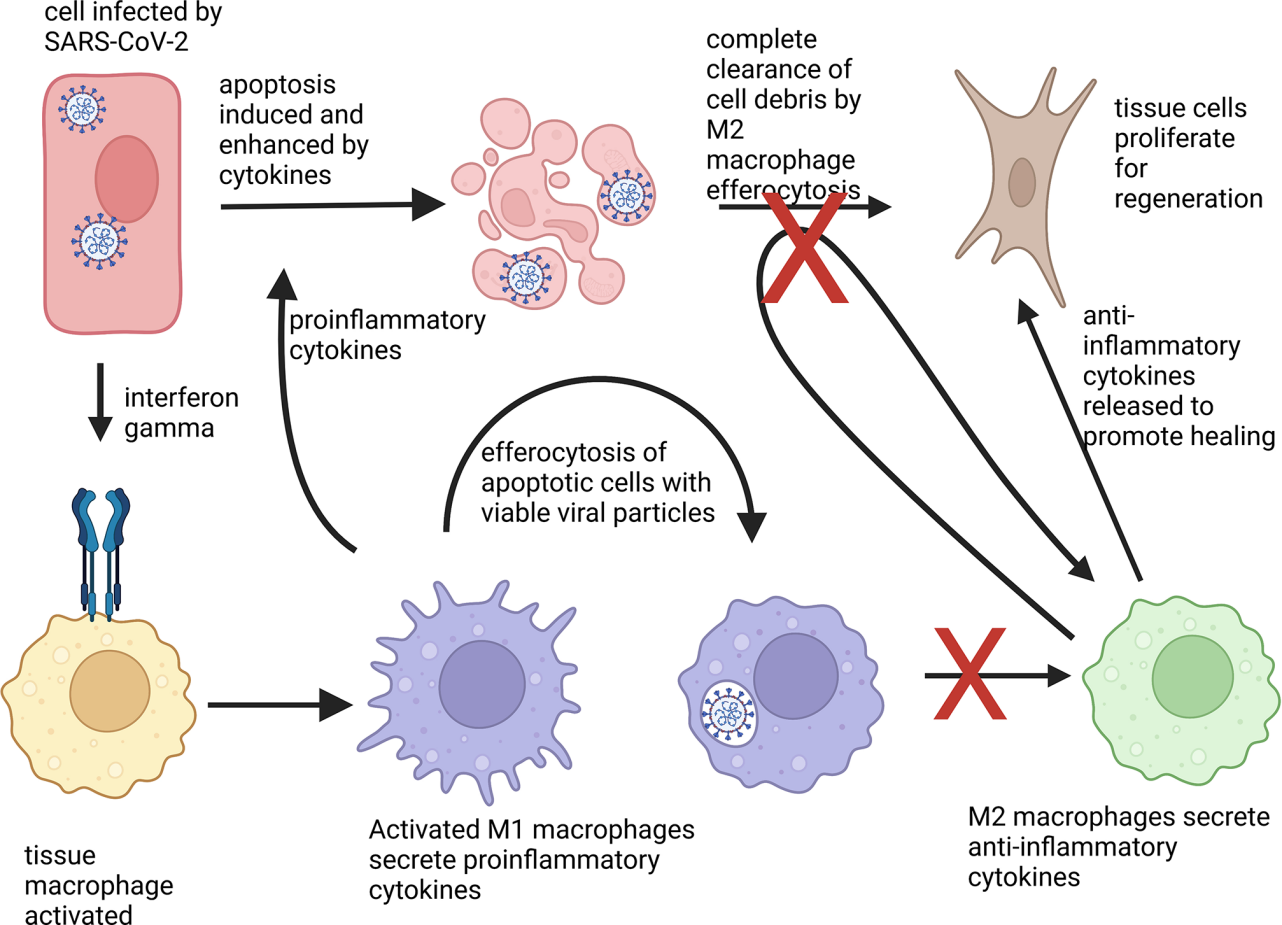
Phenotype change of macrophages in response to apoptotic cells. An epithelial cell (shown in red) attacked by SARS-CoV-2 (white) activates immune cells, known as macrophages (yellow), via the cytokine interferon gamma (blue receptor). The macrophages then differentiate into a proinflammatory state M1 (lilac) and secrete proinflammatory cytokines to activate other immune responses. M1 macrophages also help to clear cell debris and virus particles by engulfing the infected cells, a process known as efferocytosis. Normally, absorbing apoptotic (dying) cells infected with a virus causes M1 macrophages to change into M2 macrophages (green), which secrete anti-inflammatory cytokines. These in turn, stimulate fibroblasts (brown) to help regenerate damaged tissues. However, macrophages that have engulfed viable SARS-CoV-2 particles do not change into the anti-inflammatory phenotype. They also are less able to absorb pathogens and cell debris, which leads to prolonged inflammation.

Image credit: The figure was created with biorender.com.

Previous research has shown that during this transition, macrophages change their phenotype from a proinflammatory state M1 to an anti-inflammatory one, M2 (Kohno et al., 2021). It was, however, unclear how they achieve this. To find out if the same transition happens after infection with COVID-19, Salina et al. used apoptotic lung and kidney cells (that is, cells undergoing regulated cell death) containing either viable SARS-CoV-2 particles, inactivated viral particles, or sterile culture medium. They then investigated if and how engulfing apoptotic cells, a process known as efferocytosis, affects the phenotypic change of the macrophages.

The results revealed that SARS-CoV-2 prevented M1 macrophages from changing into M2 macrophages, thereby increasing the inflammatory potential of these immune cells. In the experiments, only cells infected with viable SARS-CoV-2 blocked the M1 macrophages from changing into M2 macrophages and increased the amount of proinflammatory cytokines produced, such as IL-6. Experiments with another virus species did not achieve the same outcome, suggesting that the overproduction of IL-6 may be specific to SARS-CoV-2.

Salina et al. further tested the effect of antiviral drugs targeting the transcription process of viral RNA and found that viral RNAs appear to play a significant role in preventing macrophages changing into the anti-inflammatory state. Treating macrophages with the antiviral drug Remdesivir after they had engulfed cells with viable SARS-CoV-2 reduced the production of IL-6.

These observations indicate that viral RNAs – once taken up by macrophages – arrest the immune cells to remain in the M1 phenotype, which may contribute to the cytokine storm seen in patients with COVID-19. Moreover, absorbing cells containing viable SARS-CoV-2 reduced the number of proteins responsible for recognizing apoptotic cells. This led to a build-up of cell debris and apoptotic cells.

To find out how defective efferocytosis affects the pathogenesis of COVID-19, Salina et al. stained lung tissue samples from COVID-19 patients with immunofluorescent dyes and assessed the expression of efferocytosis receptor proteins. This revealed that lung samples had a lower level of gene expression linked to efferocytosis, which lead to a reduced clearance of cell debris. It also showed that the production of cytokines was dysfunctional, suggesting that SARS-CoV-2 over-activates macrophages in the lungs. This in turn, led to severe inflammation and impaired tissue regeneration. Furthermore, the residual cell debris induced signaling molecules that activated a type of immune cells, called monocytes, to become M1 macrophages. Combined, these changes could increase inflammation even further and may prolong a dysfunctional immune response long after recovery, potentially leading to long COVID-19 syndromes.

While many questions around COVID-19 and its long-term effects warrant further research, the study of Salina et al. provides valuable insights into the complex mechanisms of cytokine storms and may open new avenues for developing treatment plans for patients with severe COVID-19 (Misra et al., 2021; Gracia-Ramos et al., 2021; Ma et al., 2022; Batlle et al., 2022; Yeung et al., 2021).
